# PGE2 binding to EP2 promotes ureteral stone expulsion by relaxing ureter via the cAMP-PKA pathway

**DOI:** 10.1186/s12894-024-01504-w

**Published:** 2024-06-08

**Authors:** Hao Su, Wenyan Zhou, Weiming Chen, Ke Yang, Meng Yang, Hu He, Cheng Qian, Dongbo Yuan, Kehua Jiang, Jianguo Zhu

**Affiliations:** 1 Department of Urology, Guizhou Provincial People’s Hospital, Guiyang, 550002 Guizhou Province China; 2 Department of Clinical Laboratory, Guizhou Provincial People’s Hospital, Guiyang, 550025 Guizhou Province China; 3 Guizhou University School of Medicine, Guiyang, 550025 Guizhou Province China; 4 Guizhou Medical University, Guiyang, 550002 Guizhou Province China; 5 Zunyi Medical University, Zunyi, 563000 Guizhou Province China

**Keywords:** PGE2, EP2, cAMP, Ureteral Calculi, Relaxation

## Abstract

**Background:**

This study investigated the relaxation effect of PGE2 on the ureter and its role in promoting calculi expulsion following calculi development.

**Methods:**

By using immunofluorescence and Western blot, we were able to locate EP receptors in the ureter. In vitro experiments assessed the impact of PGE2, receptor antagonists, and agonists on ureteral relaxation rate. We constructed a model of ureteral calculi with flowable resin and collected ureteral tissue from postoperative side of the ureter after obstruction surgery. Western blot analysis was used to determine the protein expression levels of EP receptors and the PGE2 terminal synthase mPGES-1. Additionally, PGE2 was added to smooth muscle cells to observe downstream cAMP and PKA changes.

**Results:**

The expression of EP2 and EP4 proteins in ureteral smooth muscle was verified by Western blot analysis. According to immunofluorescence, EP2 was primarily found on the cell membrane, while EP4 was found in the nucleus. In vitro, PGE2 induced concentration-dependent ureteral relaxation. Maximum diastolic rate was 70.94 ± 4.57% at a concentration of 30µM. EP2 antagonists hindered this effect, while EP4 antagonists did not. Obstructed ureters exhibited elevated mPGES-1 and EP2 protein expression (*P* < 0.01). Smooth muscle cells treated with PGE2 displayed increased cAMP and phosphorylated PKA.

**Conclusions:**

PGE2 binding to EP2 induces ureteral relaxation through the cAMP-PKA pathway. This will provide a new theoretical basis for the development of new therapeutic approaches for the use of PGE2 in the treatment of ureteral stones.

**Supplementary Information:**

The online version contains supplementary material available at 10.1186/s12894-024-01504-w.

## Background

Changes in food habits and the increased incidence of obesity are the main causes of the rising prevalence of ureteral calculi [1, 2]. Clinically, Medical Expulsive Therapy (MET) is beneficial due to its minimal trauma and complications. It is still regarded as a reasonably safe and affordable therapeutic option that may lessen patients’ financial burden. Nevertheless, there is ongoing debate regarding the therapeutic efficacy of MET. For example, the vast majority of randomised, double-blind, placebo-controlled trials did not find a benefit of MET for increased ureteral stone passage rates [3]. There are also trials that suggest MET results in a higher stone-free rate and a shorter time to stone expulsion [4]. Consequently, our aim is to explore novel pathways for stone expulsion and propose alternative modalities for MET.

Prostaglandin E2 (PGE2), a group of small-molecule lipids, is widely distributed in the human body. PGE2 serves as a vital signaling molecule and is involved in important physiological and pathological processes, such as inflammation, control of pain signals, contraction and relaxation of smooth muscles, protection of the gastric mucosa, and bone resorption and osteogenesis [5–7]. PGE2 exerts its physiological effects mainly through seven-transmembrane-domain G protein-coupled receptors, namely PTGER1(EP1), PTGER2(EP2), PTGER3(EP3), and PTGER4 (EP4)—is a crucial in vivo cell growth and regulatory element [8]. These receptors then activate other signaling pathways downstream. EP1 predominantly associates with Gq proteins to increase the concentration of Ca2 + inside the cell. EP2 and EP4 receptors primarily bind to Gs proteins, leading to an elevation in intracellular cAMP levels. EP3 binds to the Gi protein, resulting in a decrease in the quantity of intracellular cAMP via inhibiting adenylyl cyclase [9, 10]. EP receptors are sporadically present in the nucleus [11]. The primary mechanism by which PGE2 exerts its biological effects is through its interaction with receptors located on the cell membrane. As a result, most research on EP receptors has mostly concentrated on these membrane-bound receptors.

Various physiological and pathological factors can stimulate the synthesis and release of PGE2, which binds to receptors on smooth muscle and subsequently directly or indirectly regulates contraction or relaxation. PGE2 attaches to epithelial cells via EP2 and/or EP4, increasing cAMP levels and triggering adenylate cyclase to relax airway smooth muscle. This process helps to regulate asthma triggered on by exercise and bronchospasm [12, 13]. In the gastrointestinal system, The smooth muscles of the ileum, stomach, and small intestine contract upon EP3 receptor activation [5, 14]. Nevertheless, in the lower esophagus, the activation of EP2 and EP4 receptors leads to the relaxation of the circular smooth muscle in the lower esophageal sphincter (LES), whereas EP1 receptors contribute to the relaxation of the longitudinal smooth muscle [15]. Furthermore, inside the urogenital system, PGE2 is acknowledged for its influence on the relaxation or contraction of smooth muscle. PGE2 plays a role in facilitating sperm motility towards the oviduct and the movement of the fertilized egg towards the ovary in the early stages of pregnancy [16]. PGE2 demonstrates a biphasic impact during labor, initially provoking uterine contractions and subsequently promoting relaxation of the myometrium [17]. Patients exhibiting symptoms of overactive bladder (OAB) were found to have heightened amounts of PGE2 in their urine [18, 19]. Intravesical administration of PGE2 reproduced these symptoms, suggesting PGE2’s involvement in the regulation of urinary tract smooth muscle [20].

PGE2 increases cAMP levels in guinea pig ureteral tissue, resulting in relaxation of the ureter [21]. PGE2 alters the membrane potential in human ureters, hence affecting ureteral function [22]. Nevertheless, the precise mechanisms are not fully understood. Therefore, we can confidently state that PGE2 plays vital biological functions in ureteral tissue, specifically in controlling the activity of smooth muscles related to ureteral calculi. This serves as the foundation for our investigation into possible therapies for ureteral calculi.

## Materials and methods

### Cell culture

The primary cells of rabbit and human ureter smooth muscle cells were procured from Shanghai Saibaikang Biotechnology Co., Ltd. (Shanghai, China). The cells were cultivated in accordance with the recommended protocol using complete medium (10% fetal bovine serum (FBS), 100 U/mL penicillin, and 100 µg/mL streptomycin) in T25 culture flasks. Incubation was carried out in a 5% carbon dioxide environment at 37 °C with saturated humidity. Cells from passages 3 to 6 were employed in the experimental procedures.

### Animals

In our previous study, we constructed a rabbit model of ureteral calculi using flowable resin [23]. In this study, we used a sample of 25 New Zealand rabbits, comprising both males and females, with an average weight of 3.0 ± 0.5 kg and an average age of 4 ± 0.5 months. The rabbits were obtained from the Laboratory Animal Center of Guizhou Medical University, and certified under license number SCXK-2018-001. The rabbits were housed separately in cages that were kept at a temperature between 17 and 19℃. The humidity level was maintained at 40–60%. The rabbits followed a 12-hour light/dark cycle and were given a regular chow diet. The current investigation obtained approval from the Medical Ethics Committee of Guizhou Provincial People’s Hospital (Ethical Approval No: 2,017,059).

### Construction of ureteral calculi model

**Fig. 1:**
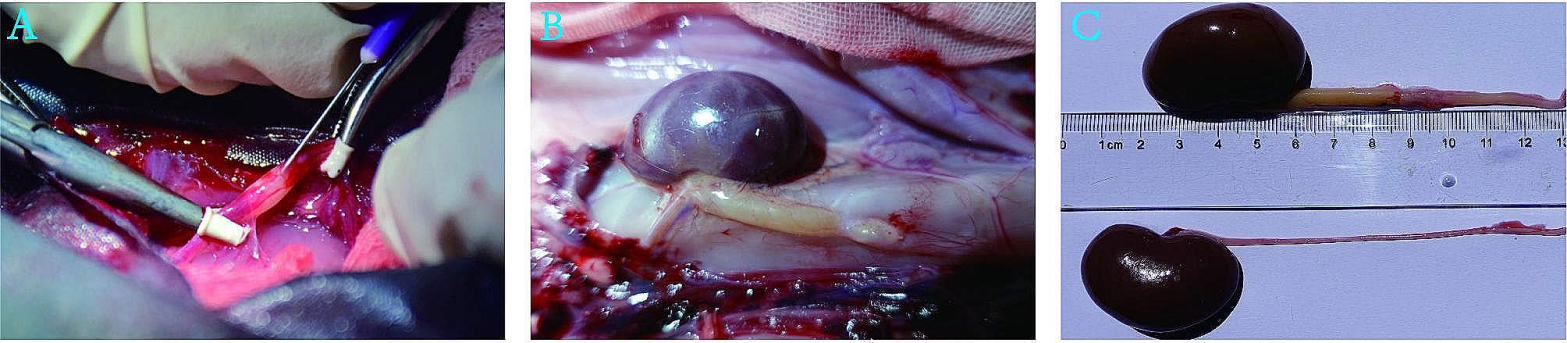
The photographs of the surgical operation and the images of the postoperative dissection show the model of the ureteral calculus. **(A)** Depiction of the surgical procedure. **(B, C)** Urinary tract images from the 7th-day group. Above is the left kidney and ureter; below is the right kidney and ureter In C

A total of twenty-five rabbits were assigned at random to five groups, with each group consisting of five rabbits. The groups were named as follows: normal group, 1st-day group, 3rd-day group, 5th-day group, and 7th-day group. The control group did not receive any treatment. We constructed a ureteral calculi model with flowable resin (FiltelKTM Z350 XT Flowable Restorative). The flowable resin is a good choice as calculi material due to its characteristics of rapid setting time, light-cured and low viscosity. The experimental groups underwent left ureteral calculus shaping according to our established procedure [23]. The rabbits in the first, third, fifth, and seventh groups were euthanized at 1, 3, 5, and 7 days after the operation, respectively. The rabbits in the control group were euthanized after 7 days. The rabbits were euthanized by administering a lethal dosage of sodium pentobarbital (30 mg/kg) through an intravenous injection. After removing the blocked tissues from the left upper ureter, the specimens were immediately placed in a pre-cooled PBS buffer. The tissues were washed with cold PBS, dried by blotting, transported to liquid nitrogen, and kept at a temperature of -80 °C. Figure 1 displays visual representations of the surgical procedure and specific urological dissections performed after the operation.

### Cell and tissue protein extraction

Cellular proteins were extracted by gently washing the cells in the T25 bottle with pre-cooled PBS, adding 200 µL of RIPA containing 1% PMSF, placing on ice, and reacting for 30 min. The mixture was then centrifuged at 4 °C at 12,000 x g for 30 min, and the supernatant was transferred to an EP tube. Tissue proteins were extracted by adding 500µL of RIPA containing 1% PMSF and placing on ice for 30 min, followed by centrifugation at 4 °C for 30 min at 12,000 x g. The resulting supernatant was transferred to an EP tube. Extracted cell and tissue proteins were assayed for protein content according to the BCA reagent instructions. The proteins were then denatured by adding the appropriate amount of loading buffer to the proteins, boiled for 10 min, cooled and stored in portions for use.

### Western blot

Equivalent proteins from different samples were isolated by 10% gel electrophoresis, after which the proteins were transferred to a PVDF membrane. The Genefist fast closure solution was applied for a duration of 10 min. EP1 (PTGER1), EP2 (PTGER2), EP3 (PTGER3), EP4 (PTGER4), mPGES-1, PKA, and p-PKA antibodies were diluted at 1:2000, while the internal reference antibodies GAPDH and β-Tubulin were diluted at 1:2000 and 1:10,000, respectively. Incubating different primary antibodies after cutting on a whole membrane results in the absence of images of adequate length. All antibodies were sourced from Affinity. Separate addition of corresponding antibodies was performed, followed by an overnight incubation at 4 °C. The membrane underwent TBST washing thrice, each for 10 min. Subsequently, goat Anti-rabbit IgG H&L (HRP) was added, and the incubation took place at room temperature for 1 h. Chemiluminescence assay kits (NCM Biotech, China) were used to visualize the protein bands in Genegenome XRQ System (Syngene, Britain). ImageJ software (v1.46r) was used to measure the density of protein bands and to convert the results into quantitative data.

### Cell immunofluorescence

Fourth-generation rabbit ureteral smooth muscle cells, exhibiting robust growth, were utilized to create a cell suspension. A total of 5 × 10^4^ cells were evenly distributed per well in a 12-well plate with a coverslip. After 24 h, once the cells had adhered to the well walls and displayed vigorous growth, the medium was discarded. A PBS rinse was performed, followed by fixation with 4% paraformaldehyde for 15 min. Subsequently, a 0.5% TritonX-100 treatment was carried out at room temperature for 20 min. Subsequently, the cells were cultured with 10% goat serum at ambient temperature for a duration of 30 min. Drops of EP1, EP2, EP3, and EP4 antibodies (diluted at 1:300, Affinity) were added to a humidified chamber and left to incubate overnight at 4°C. Following the rinse with TBST, sections were incubated with anti-rabbit IgG (Alexa Fluor 488, Servicebio, China) in the dark at room temperature for 1 h.4,6-diamino-2-phenyl indole (DAPI) (Servicebio, China) was added dropwise for 10 min to stain the nuclei. Subsequent observation and image acquisition were performed under a fluorescent microscope (Olympus, USA).

### Ureteral in vitro tension test

The rabbits were euthanized by administering a lethal dose of sodium pentobarbital through an intravenous injection. The ureter was promptly and entirely extracted into a 4 °C Krebs solution (pH7.4) pre-saturated with a gas mixture of oxygen (95% O_2_ and 5% CO_2_) with the following composition: NaCl, 118 mM; KCl, 4.7 mM; CaCl_2_, 2.5 mM; MgSO_4_, 1.2 mM; NaHCO_3_, 25.2 mM; NaH_2_PO_4_, 1.2 mM; Glucose, 11.1 mM. The surrounding fat, blood vessels, and excess tissue were microscopically removed, and the ureter was cut into small 1 cm sections. The ureter mounted horizontally on an isometric force transducer (DMT620, Denmark), filled with Krebs solution at 37 °C, and aerated with 95% O_2_ and 5% CO_2_ (Supplementary Fig. 1 in Supplementary material). The Lab Chart software, specifically the Data Monitoring Tool (DMT), was utilized to continuously record the stress in the ureter. Prior to the start of the experiment, the ureter was subjected to a static strain of 3 mN for 40 min to allow it to adapt.

1. Normal: No intervention was applied, and the tension of the ureter was examined for a duration of 1 h to monitor any alterations in its tone.
2. Measured concentration-diastolic rate curve: High potassium solution was gradually added to reach 80 mM, inducing ureteral constriction. After stabilization for 5 min, cumulative concentrations of PGE2 (ranging from 10 nM to 30 µM) were introduced. The control group was administered a comparable dosage of DMSO, and changes in ureteral tone were recorded.
3. Control: The contraction of the ureter was induced by progressively increasing the concentration of potassium solution to 80 mM, and then allowing it to stabilize for 5 min. Subsequently, 1 µM PGE2 was introduced, and the change in tension was recorded. The ureteral diastolic rate was then calculated.
4. The effect of EP receptor antagonists: Pre-incubation with 10 µM EP2 antagonist (PF-04418948) or 10 µM EP4 antagonist (L-161,982) for 10 min preceded the gradual addition of high potassium solution to 80 mM, inducing ureteral contraction. After stabilization for 5 min, 1 µM PGE2 was added, and no PGE2 was introduced to the control group. Tension changes were recorded, and the ureteral diastolic rate was calculated.
5. The effect of EP receptor agonist: Ureteral contraction was initiated by progressively introducing a high potassium solution up to a concentration of 80 mM, which was then followed by a 5-minute period overnight of stability. Afterwards, a concentration of 10 µM of the EP2 agonist (Butaprost) or the EP4 agonist (CAY10598) was introduced. Changes in tension were recorded, and the ureteral diastolic rate was calculated using the equation below.

Ureteral diastolic rate calculation: Dosed ureteral diastolic rate = (High Potassium Induced Maximum Tension - Recorded Tension After Dosing)/(High potassium Induced Maximum Tension - Eluted Tension).

### cAMP measurement

Ureteral smooth muscle cells were seeded overnight in a 96-well plate at 37 °C and subjected to the following treatments:1.Left untreated (control). 2.Cells were exposed to PGE2 (10 µM), EP2 agonist (10 µM), and EP4 agonist (10 µM), followed by an additional 2-hour incubation. 3.Cells were incubated with EP2 antagonist (10 µM) and EP4 antagonist (10 µM) for 30 min, followed by treatment with PGE2 for 2 h. Subsequently, the cells were lysed.The cAMP levels in the smooth muscle were quantified using an ELISA kit (Elabscience Biotechnology Co., Ltd, China). The standards in the ELISA kit were used to make a standard curve. Concisely, 50 µL of samples and 50 µL of biotinylated detection antibody were introduced to cAMP pre-coated wells and incubated at 37 °C for 45 min. Following the plate washing, HRP-conjugated antibodies (100 µL per well) were introduced and incubated at 37 °C for 30 min. After performing another wash, a solution of substrate (90 µL per well) was added and incubated at 37 °C for 15 min. Subsequently, a stop solution of 50 µL per well was introduced, and the optical density (OD) values at 450 nm were assessed in order to determine the cAMP levels.

### Data analysis and statistics

The experimental data were represented as the mean value plus or minus the standard deviation (Mean ± SD). Data analysis and visualization were performed using the statistical analysis program GraphPad Prism 8.0. The Student’s t-test was used to compare two groups. Multiple groups were compared by ANOVA, and post hoc comparisons were carried out by LSD method. A significance level of *p* < 0.05 denotes the presence of statistically significant differences.

## Results

### EP2 is present on ureteric smooth muscle cell membranes

**Fig. 2:**
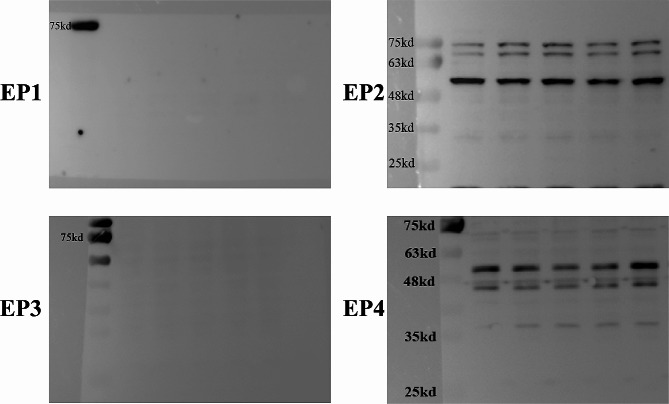
Expression of EP Proteins in Rabbit Ureteral Smooth Muscle. EP1 and EP3 proteins were not expressed. EP2(60kd) and EP4(45kd) proteins were expressed

**Fig. 3:**
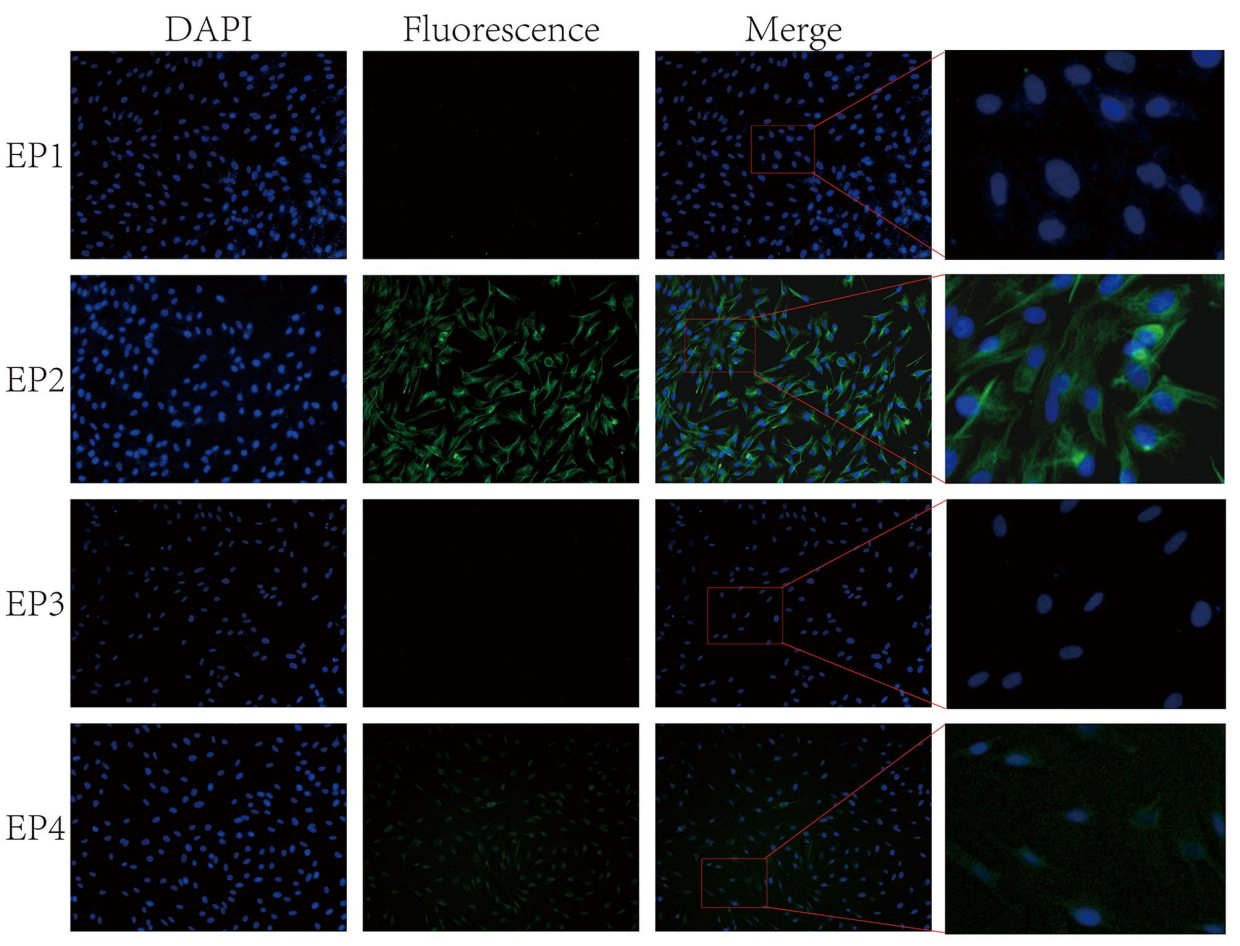
Localization of EP Receptors in Rabbit Smooth Muscle Cells. Cells were examined under a fluorescence microscope (magnification, ×200). EP1 and EP3 showed no fluorescent staining in cells. EP2 stained fluorescently in green at the cell membrane. EP4 stained fluorescently with a small amount of green in the nucleus of the cell membrane

Protein expression of EP receptors in the smooth muscle of the ureter was analyzed using Western blot analysis. EP2 was detected at the molecular weight of 60 kilodaltons, while EP4 was detected at 45 kilodaltons. Nevertheless, the expression of EP1 and EP3 proteins was absent, as seen in Fig. 2. Cell immunofluorescence was utilized to identify the cellular localization of EP expression. EP2 was detected in the cellular membrane, EP4 was detected in the nucleus, whereas EP1 and EP3 were not detected in smooth muscle cells (Fig. 3).

### In Vitro studies on the binding of PGE2 to EP2 for ureteral relaxation

**Fig. 4:**
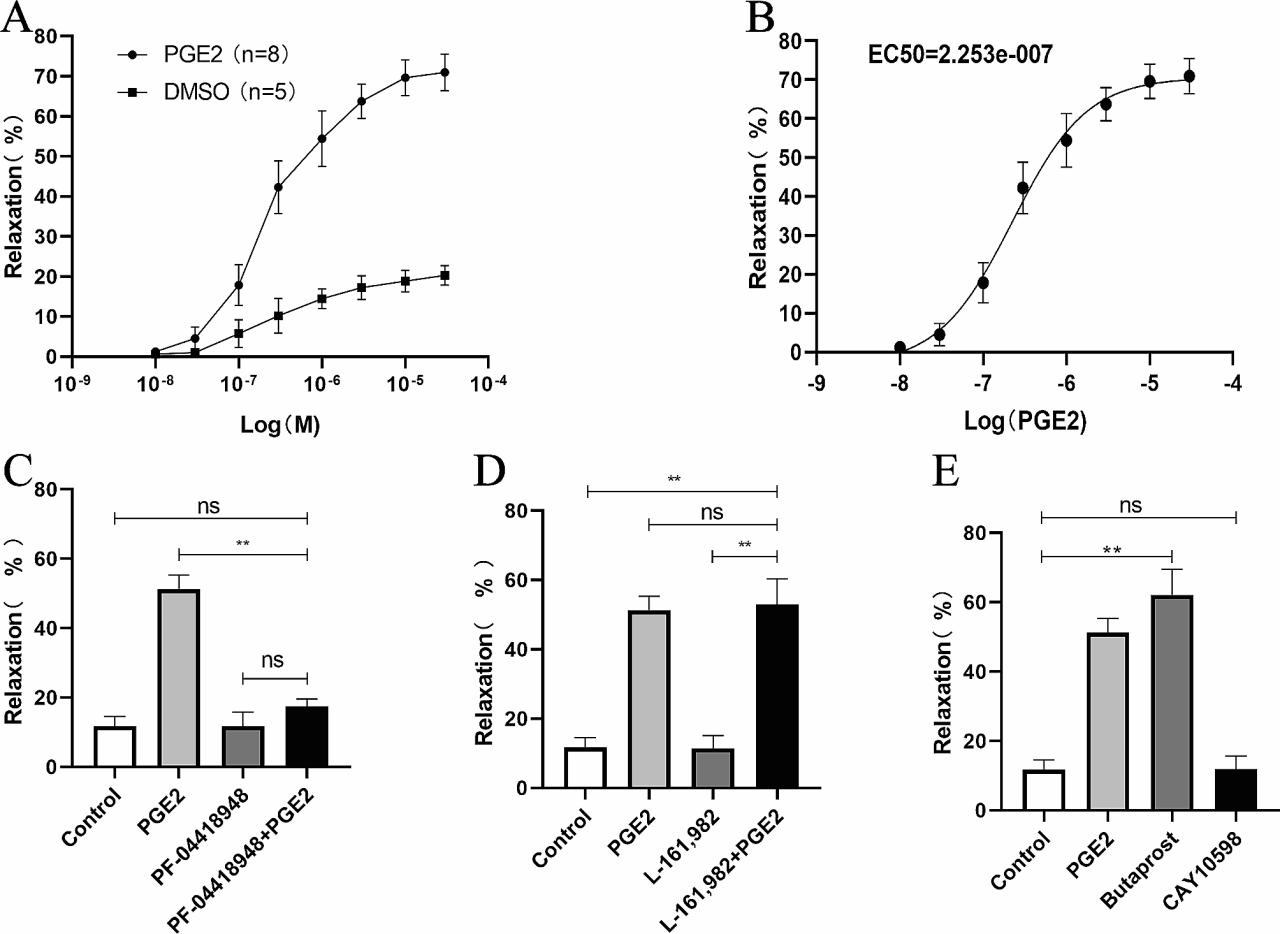
Ureteral tension responses to PGE2, EP2, and EP4 agonists or antagonists were studied in vitro. Ureteral tension responses to PGE2, EP2, and EP4 agonists or antagonists were studied in vitro. **(A)** PGE2 Concentration-Response Curves. **(B)**The effective concentration at which 50% of the maximum response is achieved for PGE2. **(C)** The PF-04418948 + PGE2 group showed a substantial disparity in comparison to the PGE2 group (*P* < 0.01). There were no significant statistical differences seen when comparing the group treated with PF-04418948 + PGE2 to the control group or the PF-04418948 group. Similarly, there were no significant statistical differences observed between the PF-04418948 group and these respective groups. **(D)** The L-161,982 + PGE2 group did not exhibit a statistically significant difference compared to the PGE2 group (*P* > 0.05). Nevertheless, the group treated with L-161,982 + PGE2 showed a substantial disparity in comparison to both the control group and the L-161,982 group (*P* < 0.01). **(E)** Butaprost demonstrated a statistically significant difference in comparison to the control group (*P* < 0.01), whereas CAY10598 did not exhibit statistically significant differences when compared to the control group (*P* > 0.05). ** *P* < 0.01; ns: not statistically significant

During in vitro investigations on ureters, it was shown that adjusting the passive tension to 3mN without any dosage treatment resulted in concentration-dependent relaxations caused by the gradual injection of exogenous PGE2. The average decrease in contraction force over a period of 1 h was 1.02 ± 0.32mN (*n* = 6), and the rate of relaxation was (11.76 ± 2.78) %. The addition of PGE2 (10nM-30µM) to ureters experiencing high potassium-induced hypertonicity resulted in an enhanced relaxation impact on the ureters as the concentration of PGE2 increased. The maximum relaxation rate was 70.94 ± 4.57% as shown in Fig. 4A. The EC50 value is 2.253 × 10^− 7^M, as shown in Fig. 4B.

Considering the EC50 value as a reference, we determined that a concentration of 1 µM of PGE2 would be suitable for the following experiment. The relaxation rate of the ureter was 51.28 ± 4.01% when exposed to a concentration of 1 µM of PGE2 (in the PGE2 group). The EP2 antagonist (PF-044189481) was added. The relaxation rate of the PF-044189481 group was measured to be 11.79 ± 3.99%. In contrast, the relaxation rate of the PF-044189481 + PGE2 group was measured to be 17.45 ± 2.10%. The PF-044189481 + PGE2 group exhibited a statistically significant difference compared to the PGE2 group (Mean Diff = 33.83%, 95%Cl(27.97, 39.69), *P* < 0.01). The differences between the PF-044189481 + PGE2 group and the control group (Mean Diff=-5.69%, 95%Cl (-11.68, 0.31), *P* > 0.05), as well as between the PF-044189481 group and the control group(Mean Diff=-5.66%, 95% Cl (-12.43, 1.10), *P* > 0.05), were not statistically significant (Fig. 4C). The EP4 antagonist, L-161,9821, was subjected to incubation. The relaxation rate of the L-161,9821 group was measured to be 11.50 ± 3.69%, while the relaxation rate of the L-161,9821 + PGE2 group was measured to be 52.99 ± 7.38%. The difference in the L-161,9821 + PGE2 group compared to the PGE2 group was not statistically significant (Mean Diff=-1.71%, 95% Cl (-8.73, 5.33), *P* > 0.05). The L-161,9821 + PGE2 group showed a statistically significant difference compared to the control (Mean Diff=-41.22%, 95% Cl (-48.49, -33.96)and L-161,9821 groups (Mean Diff=-41.49%, 95% Cl (-50.00, -32.97), *P* < 0.01) (Fig. 4D).

The EP2 agonist, Butaprost, was added. The relaxation rate of the Butaprost group was (62.08 ± 7.49)%, which showed a significant difference compared to the control group (Mean Diff=-50.32%, 95% Cl (-58.04, -42.60), *P* < 0.01). When the EP4 agonist (CAY10598) was added, the relaxation rate of the CAY10598 group was (11.82 ± 3.88)%, which showed no significant differences compared to the control group (Mean Diff=-0.05%, 95% Cl (-7.63, 7.16), *P* > 0.05) (Fig. 4E).

### EP2 protein expression in ureteral tissue

**Fig. 5:**
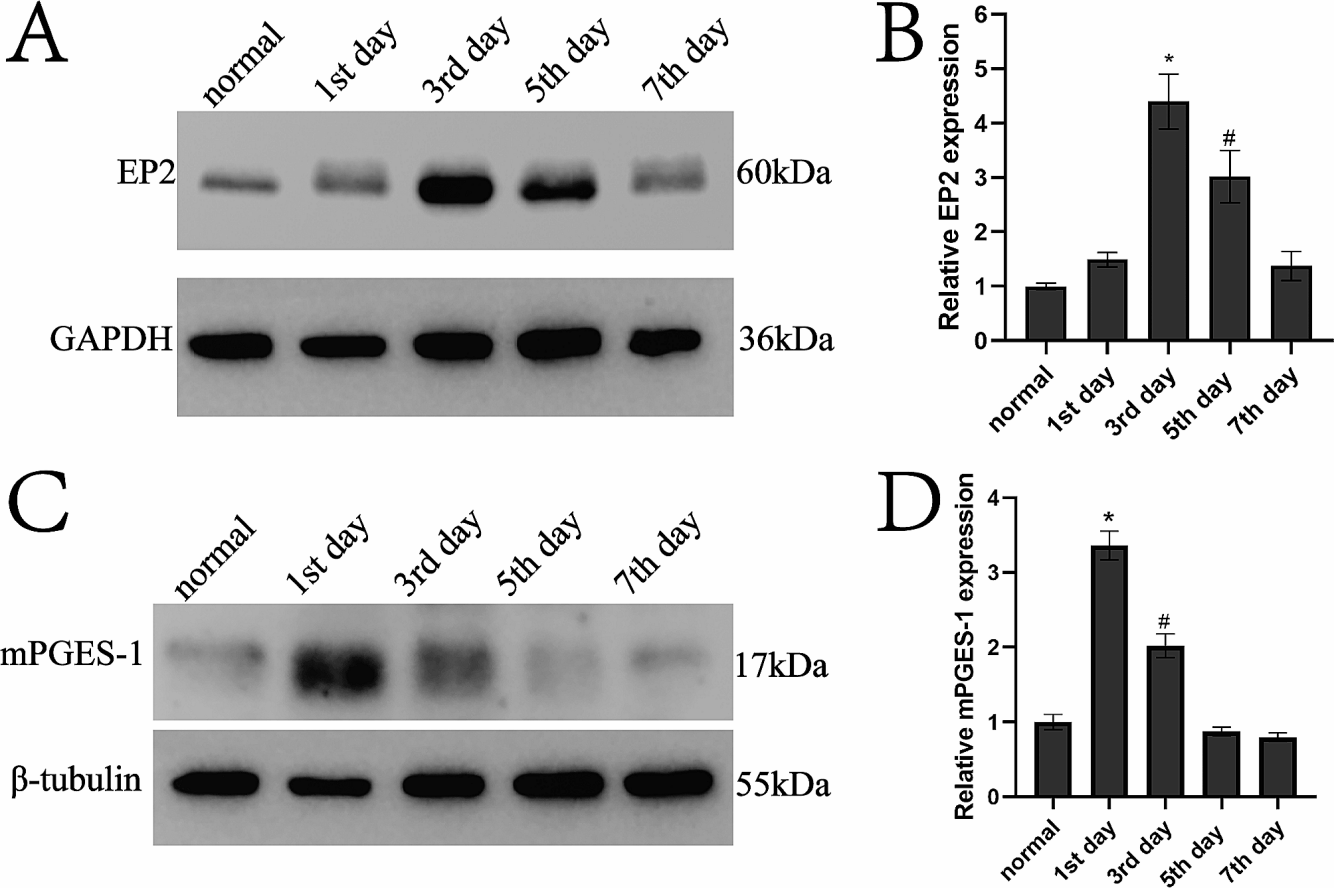
Expression of EP2 and mPGES-1 proteins in obstructed ureters at various time intervals after the formation of calculi. **(A, C)** Protein bands that serve as representatives. **(B)** Measurement of EP2 levels. The group on the third day showed statistically significant differences when compared to the other groups (*P* < 0.01). In a similar manner, the group seen on the 5th day exhibited noteworthy distinctions when compared to the other groups (*P* < 0.01). **(D)** Measurement of mPGES-1 levels. The group on the first day showed notable disparities in comparison to the other groups (*P* < 0.01). In addition, the group on the third day exhibited notable distinctions in comparison to the other groups (*P* < 0.01)

We have successfully created a model for ureteral calculi and obtained obstructed left ureters at 1, 3, 5, and 7 days after the surgery. Additionally, we collected normal rabbit ureters for the purpose of extracting proteins. The Western blot studies showed that the protein expression of EP2 was 1.00 ± 0.06 in the normal group, 1.49 ± 0.13 on the 1st day, 4.40 ± 0.51 on the 3rd day, 3.02 ± 0.48 on the 5th day, and 1.37 ± 0.27 on the 7th day. Significantly, the expression of EP2 protein reached its highest level three days after the operation, and the group on the third day showed a statistically significant difference compared to the normal (Mean Diff=-3.40, 95% Cl (-4.31, -2.48), *P* < 0.01). Similarly, the group on the 5th day exhibited a statistically significant difference when compared to the normal (Mean Diff=-2.02, 95% Cl (-2.93, -1.10), *P* < 0.01). (Figure 5A and B).

### Changes in mPGES-1 protein expression in the ureter after stone development

In pathological states, microsomal PGE2 synthase-1 (mPGES-1) functions as the main enzyme responsible for synthesizing PGE2. We evaluated variations in mPGES-1 protein expression, which serves as an indirect indicator of changes in PGE2 levels. Within this framework, we investigated the fluctuations in PGE2 concentrations in the ureters subsequent to the formation of stones, by measuring the levels of mPGES-1 in both normal and postoperative ureteral samples. The Western blot results showed that the protein expression of mPGES-1 was 1.00 ± 0.10 in the normal group, 3.36 ± 0.19 on the 1st day, 2.02 ± 0.16 on the 3rd day, 0.87 ± 0.06 on the 5th day, and 0.80 ± 0.06 on the 7th day. On the first day after surgery, the expression of mPGES-1 protein reached its highest level. The group of patients on the first day showed a significant difference compared to the normal (Mean Diff=-2.36, 95% Cl (-2.70, -2.02), *P* < 0.01). Furthermore, a statistically significant difference was noticed on the 3rd day when compared to the other groups (Mean Diff=-1.01, 95% Cl (-1.36, -0.68), *P* < 0.01) (Fig. 5C and D).

### PGE2 activates the cAMP-PKA pathway by binding to EP2 receptors

**Fig. 6:**
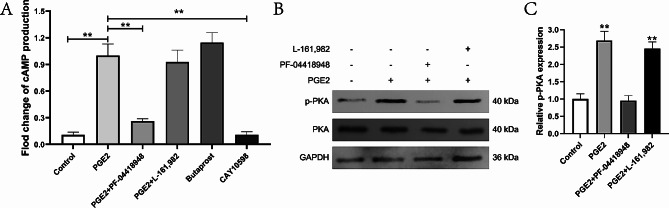
**(A)** Expression of cAMP. The PGE2 group demonstrated significant differences compared to the control group (*P* < 0.01), as well as the PGE2 + PF-04418948 group (*P* < 0.01) and the CAY10598 group (*P* < 0.01). **(B)** Representative protein band. **(C)** p-PKA Quantification. In the PGE2 group, significant differences were observed compared to the control group and the PGE2 + PF-04418948 group (*P* < 0.01). Additionally, the PGE2 + L-161,982 group showed notable distinctions in comparison to both the control group and the PGE2 + PF-04418948 group (*P* < 0.01)

PGE2 stimulates many intracellular pathways by attaching to EP2 receptors located on the cell membrane. The cAMP-PKA pathway has a crucial function in the positive regulation (relaxation) of smooth muscle. This study involved evaluating cAMP expression through the utilization of an enzyme-linked immunosorbent test (ELISA). The relative cAMP expression was 1.00 ± 0.13 in the PGE2 group, 0.16 ± 0.10 in the control group, 0.26 ± 0.03 in the PGE2 + PF-04418948 group, 0.93 ± 0.13 in the PGE2 + L-161,982 group, 1.15 ± 0.11 in the Butaprost group, and 0.11 ± 0.04 in the CAY10598 group (Fig. 6A). Additionally, we detected the expression of phosphorylated PKA (p-PKA) and PKA using western blotting. The western blot analysis indicated that the relative protein expression of p-PKA was 1.00 ± 0.15 in the control group, 2.67 ± 0.27 in the PGE2 group, 0.95 ± 0.14 in the PGE2 + PF-04418948 group, and 2.46 ± 0.18 in the PGE2 + L-161,982 group (Fig. 6B, 6 C).

## Discussions

Available evidence indicates that PGE2 has an impact on the contraction or relaxation of smooth muscles in the respiratory, digestive, and genitourinary systems [5, 12, 20]. While certain investigations have suggested that PGE2 causes relaxation of the ureter, the specific regulatory mechanism behind this effect is not well understood [21].

**Fig. 7:**
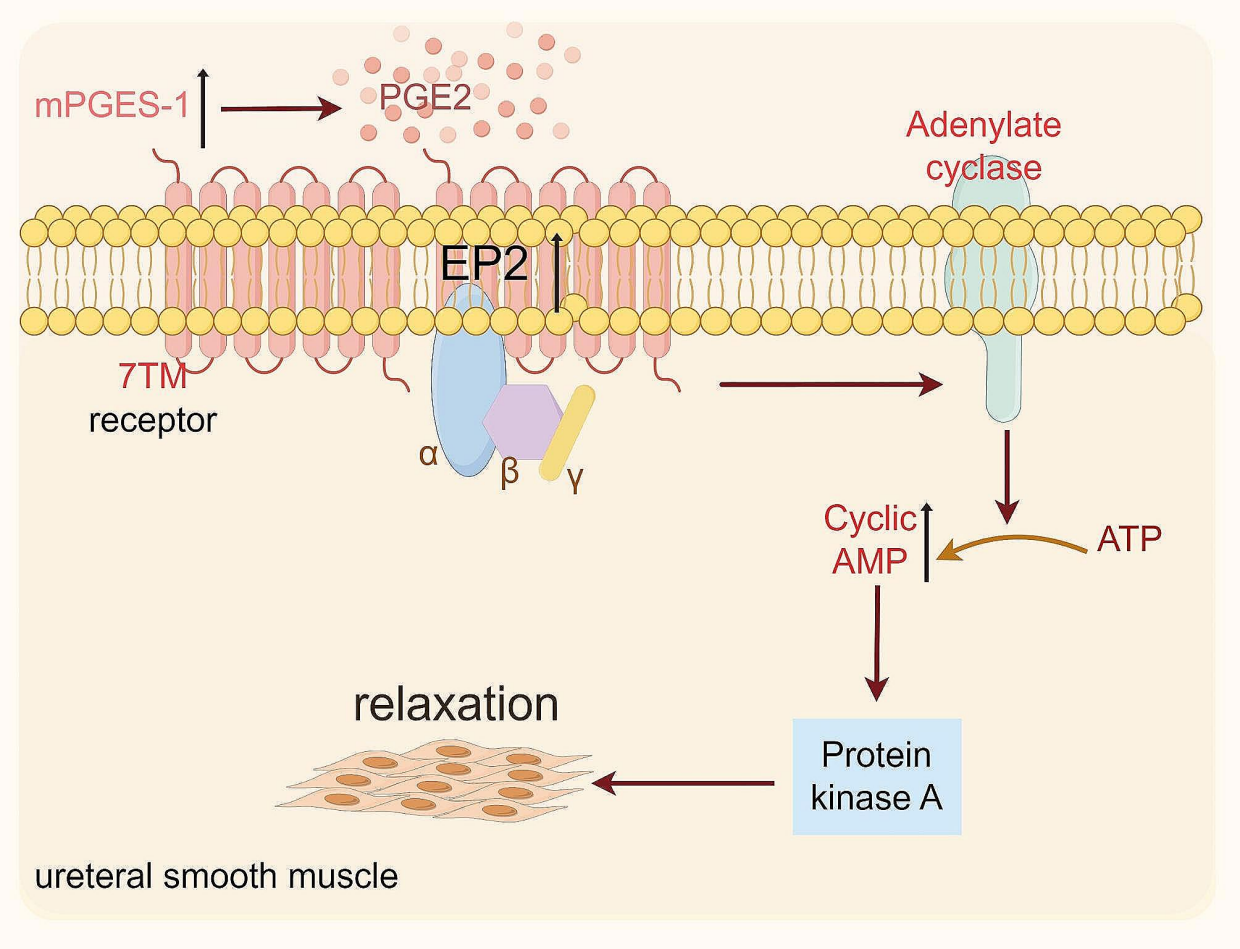
A proposed molecular mechanism through which PGE2 induces ureteral diastole to facilitate stone expulsion

Initially, it is necessary to establish the presence of EP receptors in the smooth muscle of the ureter. The protein expression of EP receptors in ureteral smooth muscle was identified by Western Blot analysis. The findings showed expression of both EP2 and EP4 proteins. The immunofluorescence staining technique was used to observe the location of EP receptors in smooth muscle cells. The findings revealed EP2 expression on the cell membrane and EP4 expression within the nuclei. Later, the impact of PGE2 on ureteral tension was examined in a controlled laboratory setting using an isolated tension assay. The findings indicated that PGE2 had the ability to induce relaxation in the ureter, aligning with the findings of a prior study [21]. Through the introduction of an EP2 or EP4 antagonist, we noticed that the EP2 antagonist effectively inhibited the relaxation effect of PGE2, however the EP4 antagonist did not have the same effect. When EP2 or EP4 agonists were included, it was shown that EP2 agonists had ureteral relaxation effects similar to those of PGE2, however EP4 agonists did not have any such effects. This experiment illustrates that PGE2 induces a state of relaxation in the ureter by specifically binding to EP2 receptors on smooth muscle, but not having the same impact on EP4 receptors. Research has verified that the binding of PGE2 to the EP2 receptor triggers the activation of PKA by causing an elevation in intracellular cAMP levels [24]. PGE2 and specific EP2 agonists induce a concentration-dependent elevation in cAMP levels, resulting in the relaxation of urethral tissue and a reduction in urethral perfusion pressure [25]. In our study, we observed a similar phenomenon in ureteric smooth muscle cells, where PGE2 specifically interacts with EP2 receptors, leading to an increase in intracellular cAMP levels and production of p-PKA. Thus, we hypothesize that PGE2 induces a relaxation of the smooth muscle by binding to EP2 receptors, thereby activating the cAMP-PKA pathway, as depicted in Fig. 7. We also found similar EP receptor expression in human ureteral smooth muscle cells (supplementary Fig. 2). However, limited by the availability of human ureteral tissue specimens, we did not obtain human ureteral responses to PGE2.

The occurrence of ureteral calculi has been steadily rising in recent years [26]. Gaining a more profound comprehension of the regulation of ureteral contraction and relaxation is crucial for maximizing the effectiveness of ureteral stone therapies. We fabricated models of ureteral calculi and collected ureteral tissue from the blocked side. By doing Western blot analysis, we noticed a gradual and time-dependent rise in the expression of EP2 protein in the ureter on the obstructed side. The peak of EP2 was observed on the third day after the onset of calculi, followed by a subsequent drop. On the seventh day after the surgery, there was no noticeable distinction when compared to a healthy ureter. This suggests that the body may enhance ureteral relaxation by upregulating EP2 expression, which facilitates PGE2 binding in response to ureteral calculi formation.

In vivo, three primary enzymes—membrane prostaglandin E2 synthase (mPGES)-1, mPGES-2, and cytosolic prostaglandin E2 synthase (cPGES)—are responsible for the production of PGE2 [27, 28]. In pathological states such as inflammation, discomfort, and fever, mPGES-1 is the main producer of PGE2 [29]. Consequently, we examined the expression of mPGES-1 at various days post calculi occurrence to indirectly assess changes in PGE2 content. The results revealed that mPGES-1 was highest in the ureter of the obstructed side on the first postoperative day, gradually decreasing thereafter. This pattern aligns with the pathological changes following ureteral calculi development. When ureteral calculi become trapped, the stones cause friction against the lining of the ureter, leading to inflammation. These factors result in an increase in the expression of mPGES-1, which in turn leads to an augmentation of PGE2 production. Therefore, we propose that the increased levels of PGE2 following the formation of ureteral calculi enhance the ability to bond with EP2, which contributes to the favorable factor for the expulsion of the stone.

In this study, we observed a noteworthy phenomenon in the ureteral calculi model. The expressions of certain proteins increased in the postoperative period. However, by the seventh postoperative day, the relative expression of these proteins did not exhibit a significant difference compared to the normal group. Additionally, our clinical observations revealed a compelling pattern. Failure to expel stones within the initial 7 days following ureteral calculi development correlated with a decreased likelihood of later expulsion. Therefore, we speculate that EP2 and mPGES-1 proteins are increased in the 7 days after stone occurrence compared with normal tissue. Ureteral relaxation promotes the expulsion of calculi. However, after 7 days, the proteins expressed decreased, ureteral relaxation decreased, and stone expulsion was difficult. These results suggest the need to explore these associations further.

Alpha blockers are widely used in the medical treatment of ureteral stones [30]. By binding to alpha receptors, it reduces ureteral constriction and promotes spontaneous stone expulsion. In our study, the inflammation produced by stones was in a controlled state of the organism. In contrast, the functional strength of PGE2 at physiological concentrations is, in any case, at a controllable level for the organism. Until there is a definitive functional assessment, it may serve more as a favourable factor for stone expulsion than as a determinant. Of course, we do hope that it will be found in future functional studies that its dosage effect produces a significant stone-excreting function. At least a unique efficacy for stone expulsion or a favourable outcome in terms of functional exogenous response.

We acknowledge there are limitations in this study. First, the use of an animal model has notable limitations for the study of any disease state. In our study, there are limitations of using animal models to infer human physiology or the potential variability in EP receptor expression among individuals. Second, the use of open wound implantation material in rabbits in our study may also have brought about a more severe stress reaction affecting the results of the study. Thirdly, we did not directly observe the phenomenon of ureteral stone discharge. Fourth, the absence of a direct comparison between the effects observed in the rabbit model and human tissue samples. This needs to be further verified experimentally and clinically. At the same time, we just propose that the PGE2-EP2-cAMP-PKA pathway could be a potential therapeutic target for the treatment of ureteral stones. However, the pathway also has potential risks. In the next part of our study, we will explore the safety or side effects of manipulating this pathway.

## Conclusion

In ureteral tissue, PGE2 binds to EP2, activating the cAMP-PKA pathway and resulting in ureteral relaxation. Targeting this pathway may provide a new theoretical basis for the development of new avenues for non-invasive treatment of ureteral stones.

### Electronic supplementary material

Below is the link to the electronic supplementary material.

Supplementary Material 1

## Data Availability

The images and datasets used in the current study is available from the corresponding author on reasonable request.
