# Supplementary material for: PGE2 binding to EP2 promotes ureteral stone expulsion by relaxing ureter via the cAMP-PKA pathway
Source: BMC Urol. 2024 Jun 8;24:117. doi: 10.1186/s12894-024-01504-w (PMC11161962; doi:10.1186/s12894-024-01504-w)
Supplement: Supplementary file 1 — Supplementary Material 1 [file 12894_2024_1504_MOESM1_ESM.docx]

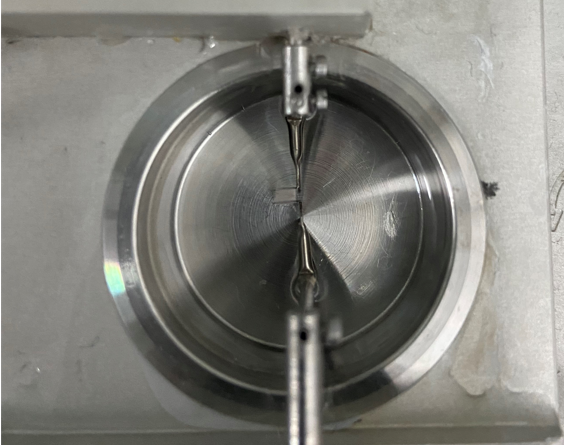


Supplementary Fig 1. Picture of ureteral tissue fastened in a DMT620 instrument.


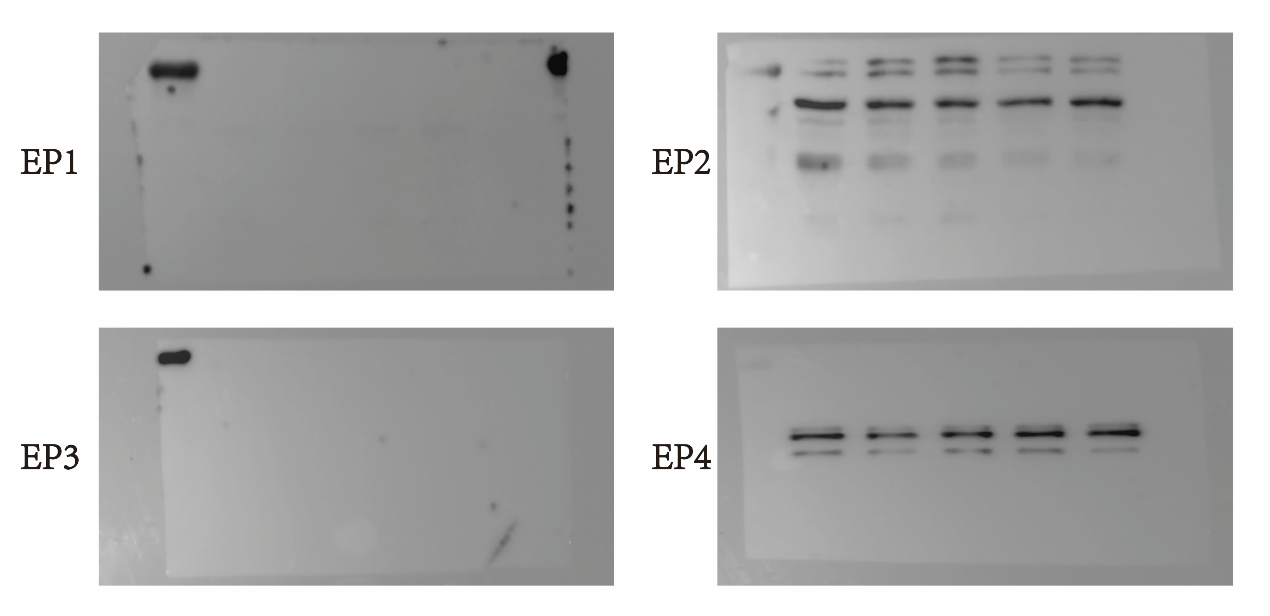


Supplementary Fig 2. Expression of EP receptor in human ureteral smooth muscle cells.

**Full-length gels and blots**

Figure 2


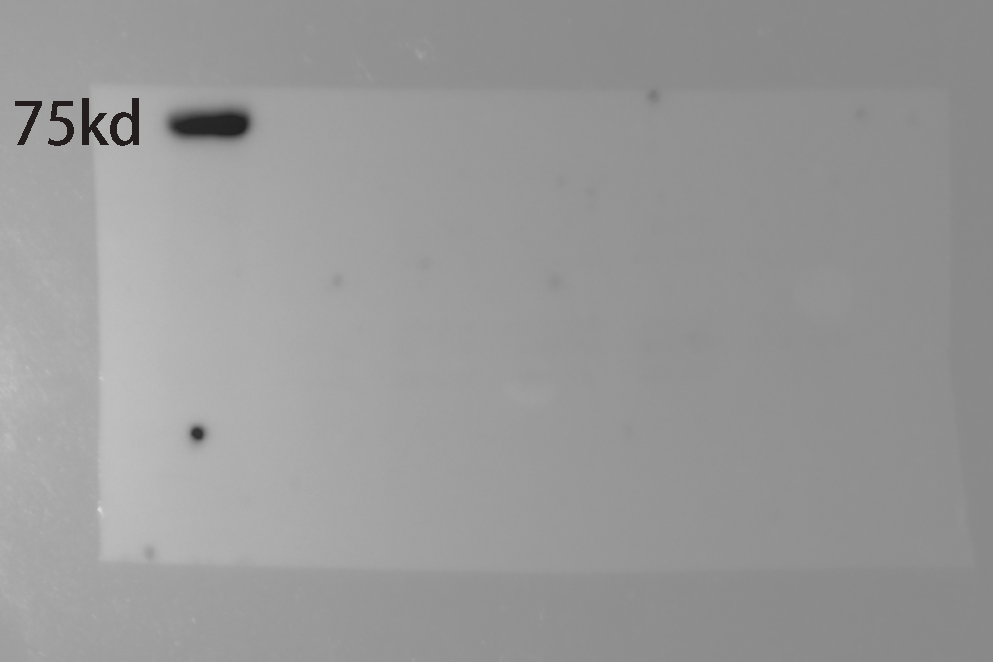


EP1


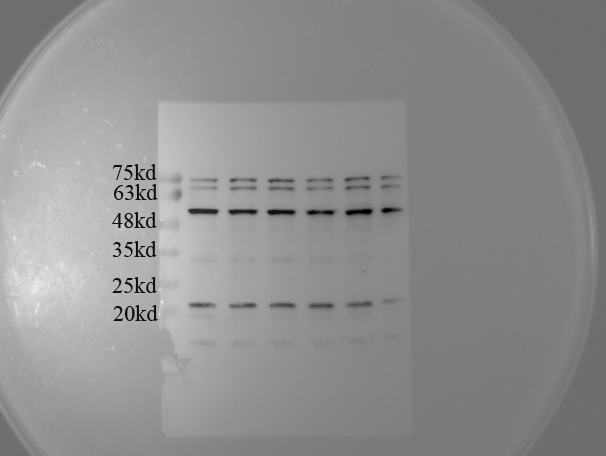


EP2


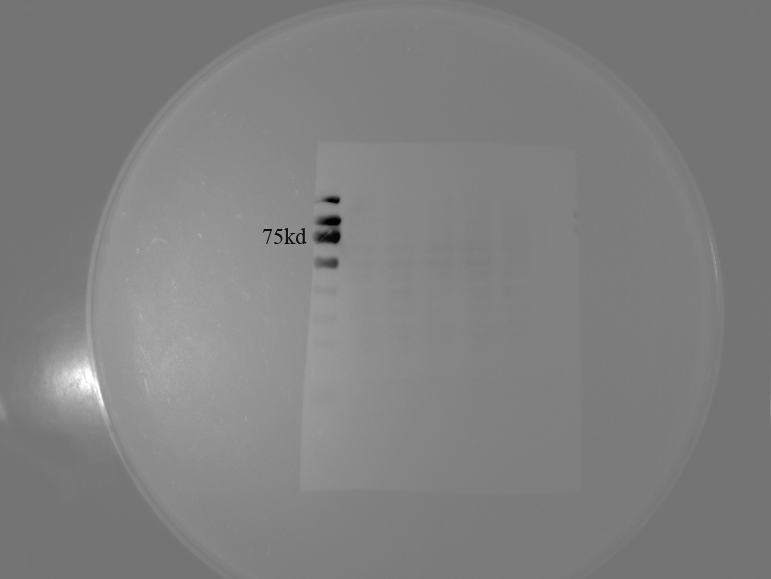


EP3


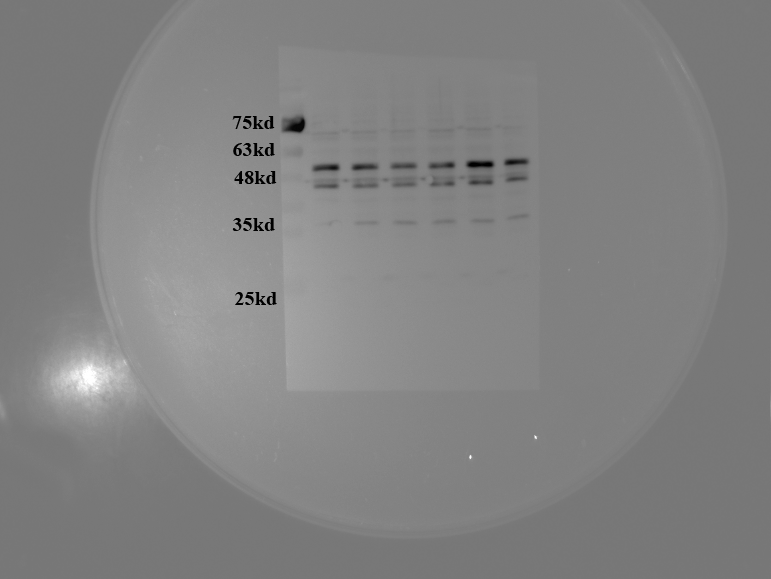


EP4

Figure 5


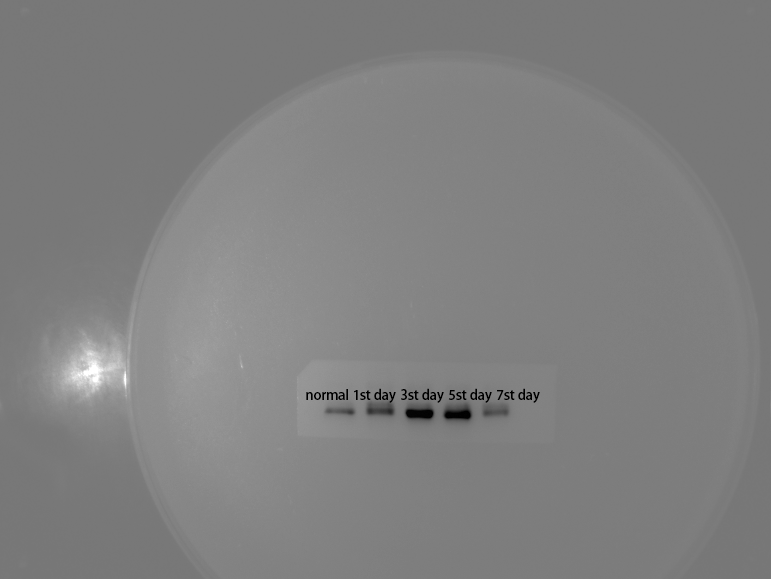


2A EP2


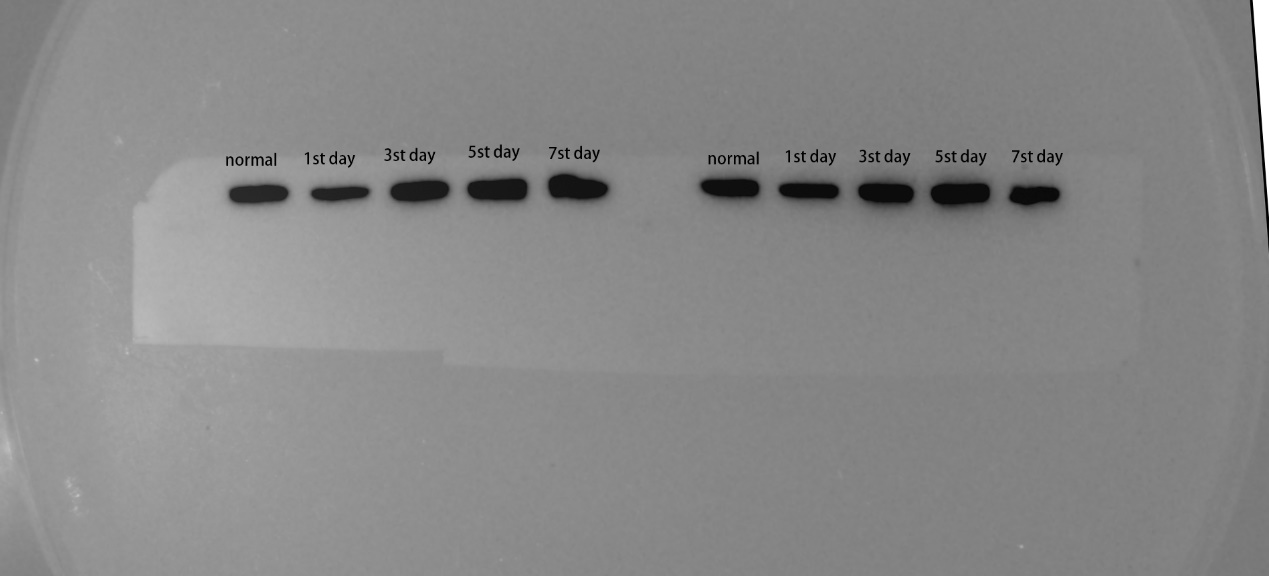


2A GAPDH The right side is the strip shown in the figure


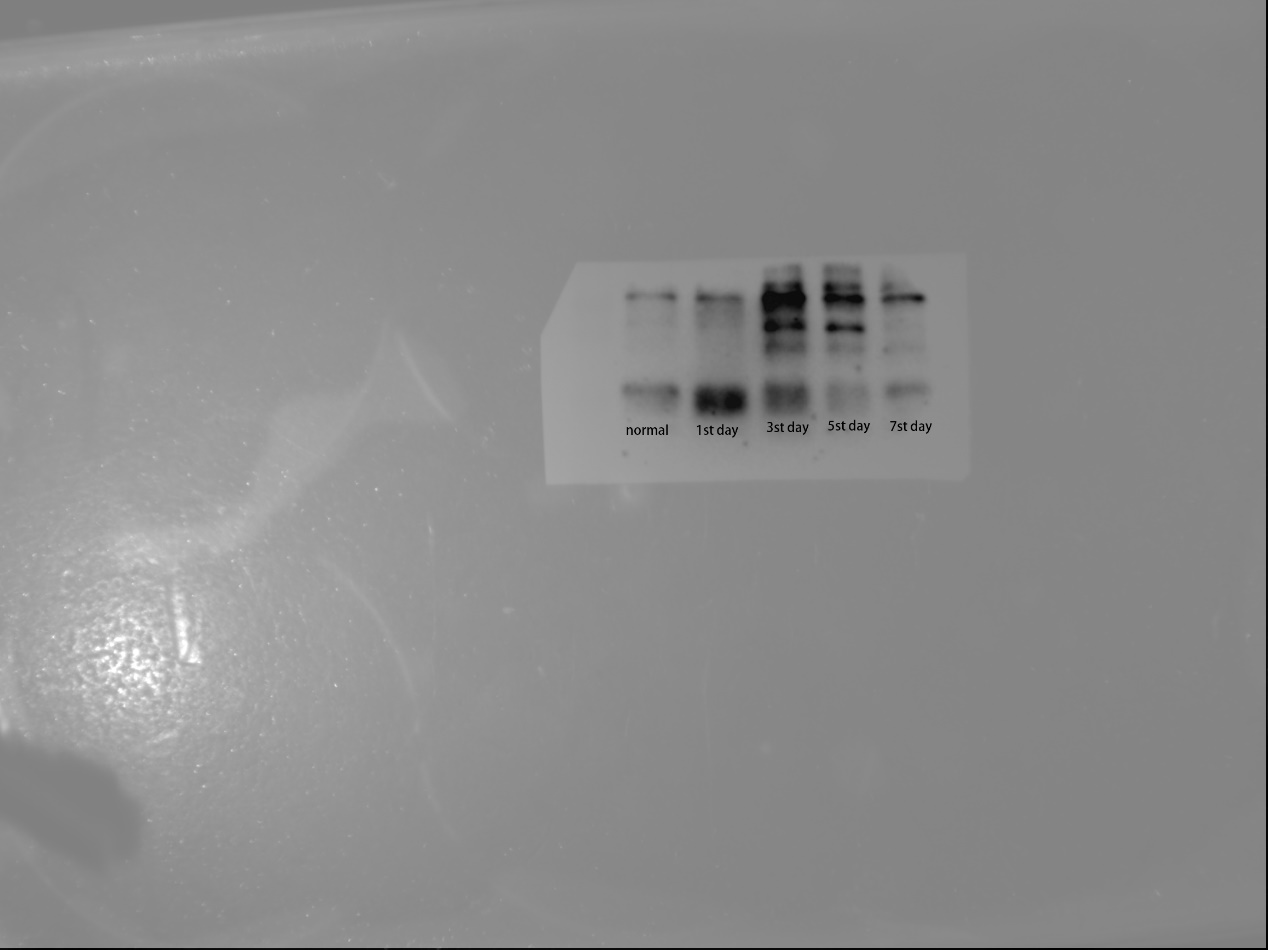


2C mPGES-1 The molecular weight of mPGES-1 is 17KD, so we considered the following band to be mPGES-1.


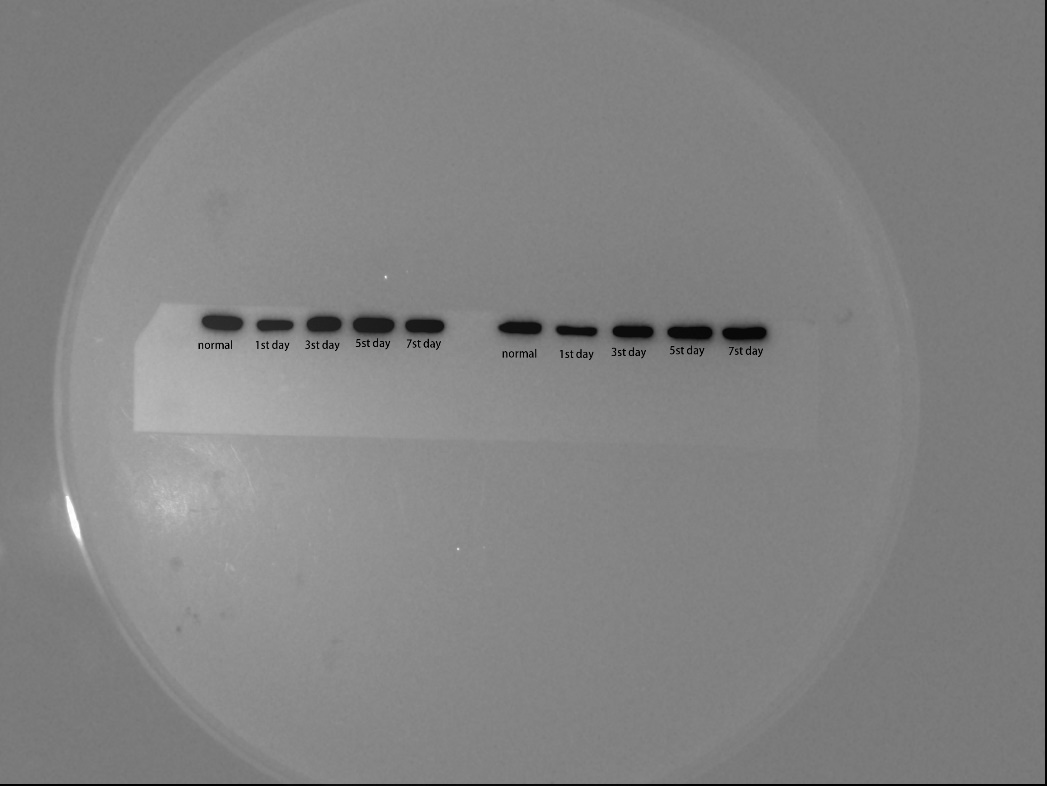


2C β-tubulin The right side is the strip shown in the figure

Figure 6


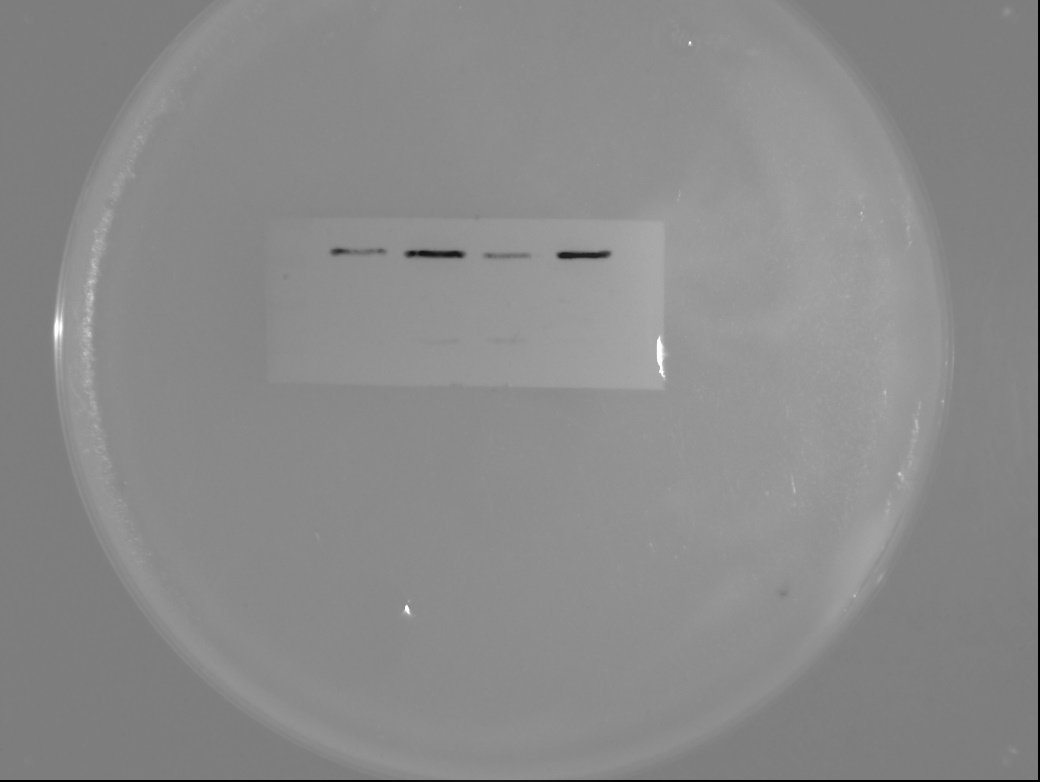


6B p-PKA


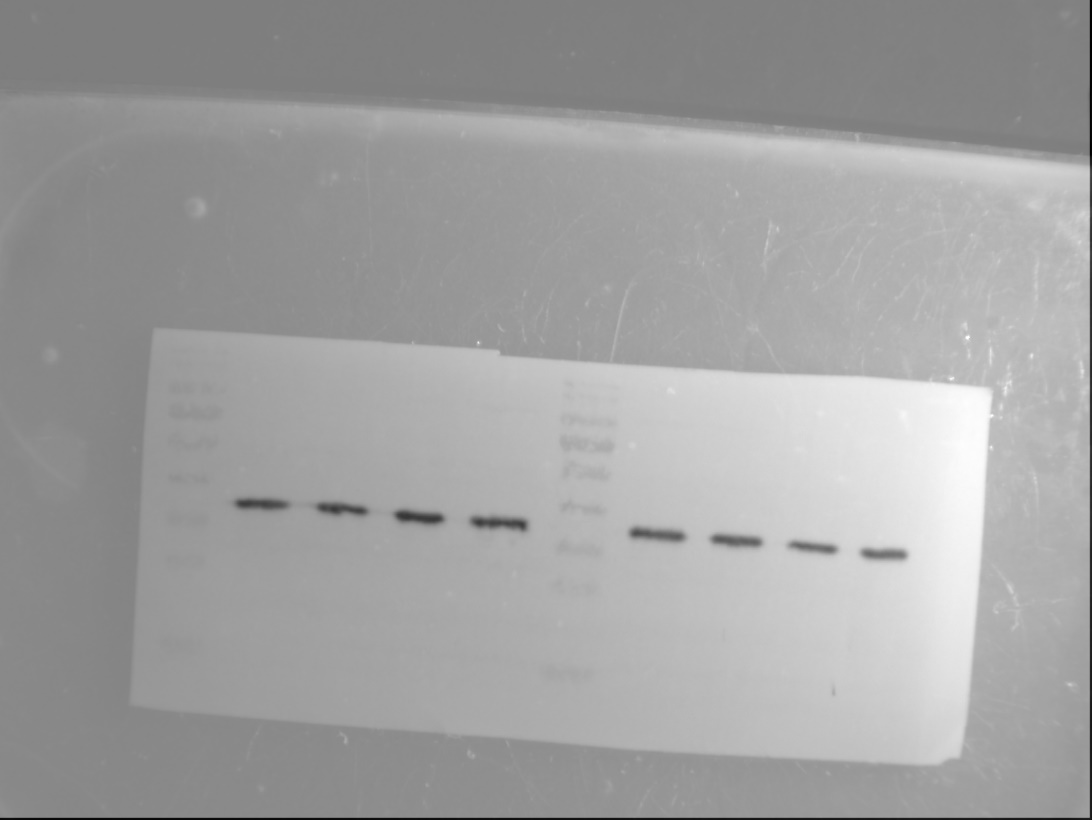


6B PKA


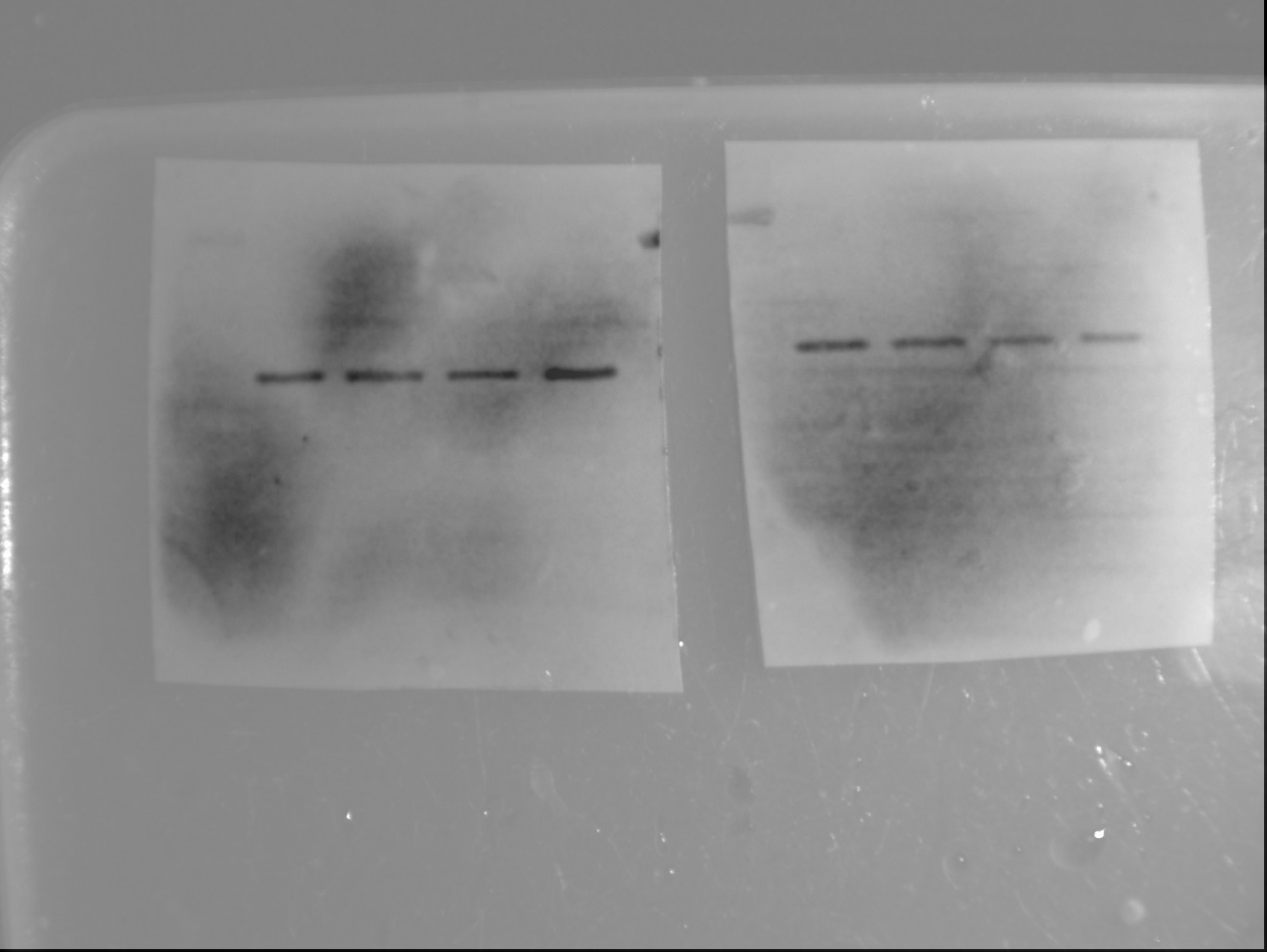


6B GAPDH The left side is the strip shown in the figure
